# Mitochondria: between aging, frailty and sarcopenia

**DOI:** 10.18632/aging.204998

**Published:** 2023-08-22

**Authors:** Annalisa Davin, Riccardo Rocco Ferrari, Orietta Pansarasa

**Affiliations:** 1 Cellular Models and Neuroepigenetics Unit, IRCCS Mondino Foundation, Pavia, 27100, Italy; 2 Laboratory of Neurobiology and Neurogenetics, Golgi-Cenci Foundation, Abbiategrasso, Italy

**Keywords:** frailty, sarcopenia, mitochondrial dysfunction, mtDNA, biomarkers

On November 15, 2022, the total world population exceed the 8 billion milestone of with a steadily increase in life expectancy and aging [United Nations, 2020]. Parallel to aging, frailty is becoming a crucial topic because of its close association with older age.

Frailty is a multi-dimensional syndrome characterized by a decline in reserve and resistance to stressors across multiple physiological systems, strictly related to adverse outcomes: falls, fractures, difficulties in daily living activities, disabilities, hospitalizations and mortality. Two approaches are primarily adopted to define physical frailty: the deficit model that consists of adding together an individual’s number of impairments and conditions to create a Frailty Index and the other model that originally defined a specific physical phenotype investigating 5 possible components (weight loss, exhaustion, weakness, slowness, and reduced physical activity) [1].

A decline in muscle mass, function and strength, otherwise defined sarcopenia, increase the risk of incurring in health-related negative outcomes. Taking into account that sarcopenia is a clinical condition present in half of people over 80, it is evident that there is a remarkable overlap with frailty so as to be defined physical frailty and sarcopenia [2].

Being frailty a dynamic condition, it is possible to hypothesize socio-economic or drug preventative interventions to tackle age-associate frailty and promote resilience.

In this scenario, the discovery of innovative biomarkers can be useful to orientate the interventions design and find the better window-time of their efficacy. Nevertheless, considering the concept developed from Junius-Walker at al. (2018), that frailty probably derived from a based complex systems integrating the interplay of biological and non-biological factors [3], it is crucial to consider if some biological events are pure aging-associated changes, independent from other life events or are specific frailty determinants.

A key point in the identification of potential biomarkers of frailty is the discovery of the hallmarks of aging. Among these, mitochondrial dysfunctions are central in the regulation of the aging process. Age-related impairment in mitochondrial function has been largely investigated assuming it may underlie multiple biological changes (decreased ATP production and energy reserves, increased free radicals production, altered rates of apoptosis and mitophagy) that increase vulnerability, functional and cognitive decline and also mortality [4].

Older adults with physical frailty and sarcopenia showed an increased level of exosomes characterized by the presence of lower levels of mitochondrial components ATP5A (complex V), NDUFS3 (complex 1) and SDHB (complex II). This scenario may reflect the cellular need to extrude dysfunctional mitochondria and, therefore, the presence of impaired mitochondrial quality control processes or a mitochondrial damage too severe to generate and release mitochondrial-derived vesicles [2]. Furthermore, a multi-marker study based on the adoption of an innovative SO-Cov-Sel-LDA analytical approach, found that the combination of five biological relevant factors among systemic inflammation, metabolic derangements and circulating mitochondrial-derived vesicles can discriminate between older adults with or without physical frailty and sarcopenia [5].

Another crucial point, is the importance of the mitochondrial DNA (mtDNA) during the aging process, with some mtDNA variants that modulate the risk of several age-associated diseases and syndromes including frailty as well as mtDNA copy number (considered a marker of mitochondrial replication and cellular energy reserve) [4]. Ashar and colleagues demonstrated the association between a lower level of mtDNA copy number and increased age, sex (male gender) and frailty in two large multi-ethnic cohorts. These findings are in line with other studies reporting a protective effect of high mtDNA copy number with respect to survival and increased energy reserve [4].

All these results support the existence of a “citokinome” in frail older adults, in which the release of noxious material from injured cells (i.e., damage-associated molecular patterns (DAMPs)) triggers caspase-1 activation and the secretion of pro-inflammatory cytokines. This setting is not exclusively specific to the physical frailty combined with sarcopenia, but it is a converging mechanism common to many age-related diseases including neurodegenerative disorders, but also to the condition of HIV infection, a model of accelerated and exacerbated aging [2].

**Figure 1:**
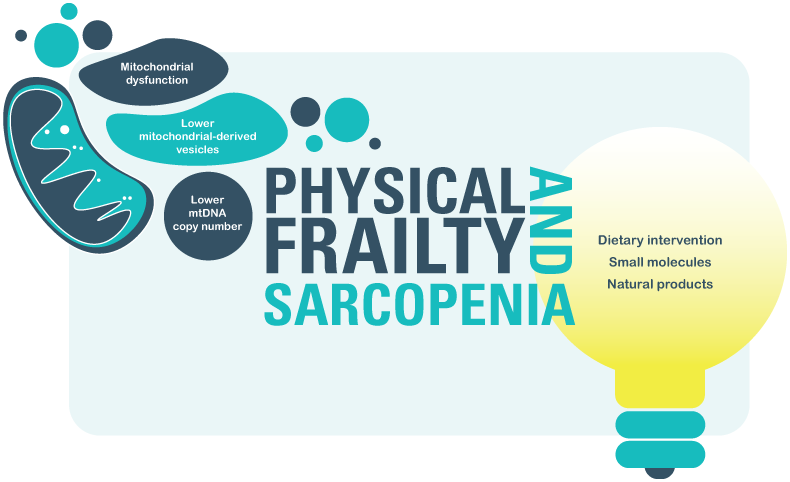
Involvement of mitochondria in physical frailty and sarcopenia and possible restorative treatments.

The elucidation and involvement of mitochondria, mainly of their dysfunction, in physical frailty and sarcopenia could represent a crucial step to test new and encouraging interventions to increase resilience by restoring mitochondrial function. Today, the scientific community extensively uses small-molecules, natural products and dietary interventions to modulate mitochondrial function, senescence, and nutrient-sensing metabolic signals to improve cellular aging and age-related diseases. However, despite the tremendous efforts, most of our knowledge about the therapeutic prospects of natural products and dietary supplements comes from basic studies utilizing native laboratory-adapted species, therefore this promising way needs to be further deepened with ad hoc studies on large human populations [6]. The other sparkling weapon of the possible restorative treatments of mitochondrial dysfunctions is the transplant of younger mitochondria. This treatment has been tested on animal models with compelling results in the immediate time but with a loss of efficacy within a month, thus suggesting a potential benefit for acute rather than chronic condition (Figure 1). Further future researches, optimization and technology advances are required to make this treatment effective and therefore useful in age-related diseases [7]. To conclude, even if considerable effort is still certainly required to face the frailty syndrome, mitochondria may potentially offer an important point of departure for its management.
